# The worm in the world and the world in the worm

**DOI:** 10.1186/1741-7007-10-57

**Published:** 2012-06-25

**Authors:** Mark Blaxter, Dee R Denver

**Affiliations:** 1 Institute of Evolutionary Biology, University of Edinburgh, Edinburgh EH9 3JT, UK; 2 Department of Zoology, Center for Genome Research and Biocomputing, Oregon State University, 3029 Cordley Hall, Corvallis, OR 97331, USA

## Abstract

*Caenorhabditis elegans *is a preeminent model organism, but the natural ecology of this nematode has been elusive. A four-year survey of French orchards published in *BMC Biology *reveals thriving populations of *C. elegans *(and *Caenorhabditis briggsae*) in rotting fruit and plant stems. Rather than being simply a 'soil nematode', *C. elegans *appears to be a 'plant-rot nematode'. These studies signal a growing interest in the integrated genomics and ecology of these tractable animals.

See research article http://www.biomedcentral.com/1741-7007/10/59

## Commentary

*Caenorhabditis elegans*, the free-living nematode tamed as a new model organism by Sydney Brenner in the 1960s [1], has become a keystone species in the ecology of scientific knowledge. The ease with which *C. elegans *can be grown, manipulated and observed has driven biomedical research into new areas and 'the worm' has been a silent collaborator in three Nobel prizes, and thousands of research articles over the past 50 years. While primarily chosen because of the ease of genetic analysis, interest in *C. elegans *was redoubled when it became the first animal to have its whole genome sequenced [2]. The genome revealed much about the basic machinery of being an animal, and the specifics of being a nematode. One of the greatest surprises was the discovery of over 1,280 putative chemoreceptor genes [3]. This exuberant repertoire (even dogs have only approximately 1,200 olfactory and chemoreceptor genes) suggests that the nematodes' wild environment must be extraordinarily complex. However, the true ecology of *C. elegans *has remained enigmatic. In the laboratory it is clearly a boom-and-bust 'r-strategist' - a single self-fertilizing hermaphrodite (with an occasional rare male), given enough agar plates, *Escherichia coli *food and willing lab assistants, could produce over a billion great-granddaughters in a month. But where does it live, feed and reproduce in the wild? And how has its wild environment shaped the biology now explored in high-throughput investigations in labs worldwide? Marie-Anne Félix and Fabien Duveau report in *BMC Biology *[4] new findings from nematode populations in orchards near Paris that provide some answers to these questions.

## A global nematode

While often called a 'soil nematode', *C. elegans *has rarely been isolated from soils [5]. Sydney Brenner's *C. elegans*, the iconic N2 strain, came from mushroom compost in Bristol, UK. Like many related nematodes, *C. elegans *can enter a facultative diapause stage, the dauer, in response to crowding and lack of food. A modified third stage larva, the dauer can survive without feeding for months (compared to the few days of a normal nematode's life), and is resistant to desiccation and other environmental stresses. Dauers can be isolated from compost heaps and other environments rich in rotting vegetation and are commonly found associated with arthropods (woodlice and pillbugs) and molluscs (snails and slugs). It is likely that these invertebrates are used as transport hosts for dispersal to new food sources. Dauers can be isolated from compost heaps and other environments rich in rotting vegetation and are commonly found associated with arthropods (woodlice and pillbugs) and molluscs (snails and slugs). It is likely that these invertebrates are used as transport hosts for dispersal to new food sources. New wild strains can be established from dauers, as the vast majority of individuals will be hermaphrodites capable of founding a dynasty. While useful, dauer-derived isolates are uninformative of the feeding ecology of the species, the dynamics of the lifecycle or mating strategies in the wild.

Several hundred *C. elegans *strains have been isolated from all continents save Antarctica, and even from isolated islands such as Hawai'i, largely from compost or transport hosts. There is little geographical structure to global *C. elegans *populations, suggesting extensive dispersal, likely through human agency. Genomic analyses of natural polymorphism patterns in these global strains reveal that while some (for example, those from Hawai'i) are very different from all others, most share large blocks of their chromosomes in a striking mosaic pattern [6]. This is unexpected, and suggests that the pattern of lifecycle history of the species likely includes frequent events of isolation by distance and founding of new populations by small numbers of related nematodes, and less common events of crossing between distinct evolutionary lineages. This study also revealed strong selective sweep events in *C. elegans*, presumably driven by selection acting on one major, beneficial mutation that drags along large chromosome blocks with it. But what is the ecological context of beneficial mutations in *C. elegans*? Where does the rare sex happen? Where are the breeding populations of *C. elegans *to be found? Until these questions are answered, the growing program of research on the population genetics and evolutionary genomics of *C. elegans *[7] will lack power and reach.

## Windfall apples, full of worms

Few researchers would need much persuading to take a break from the lab or office on a late summer afternoon, and picnic in an idyllic, ancient orchard in the quiet countryside near Paris. What motivated Marie-Anne Félix's visits to the orchards of Orsay and Santeuil, however, was the opportunity to track for the first time robust, proliferating populations of *C. elegans *(and the related *Caenorhabditis briggsae*) in the wild. She and her lab team returned to survey the orchards for four seasons, and also examined additional rotting fruit and plants for *Caenorhabditis *across France, and are now able to report on the ecology of these populations [4].

*C. elegans *was found in rotting fruits of many kinds, and also in the rotting stems of herbaceous plants. It was frequently found with *C. briggsae*, and both species could be found in the same fruit. The nematodes were surprisingly common (20% of rotting apples from Orsay harbored nematodes) and showed reproducible seasonal changes in abundance. The rotting fruit and stems often contained many hundred animals, and all life stages were present, including rare males. As might be predicted from laboratory knowledge of the temperature preferences of the two species, *C. briggsae *was commoner in the warmth of the summer, and *C. elegans *in the cooler autumn. Félix and Duveau carried out competition experiments to show that indeed Orsay and Santeuil *C. briggsae *outcompeted co-isolated *C. elegans *at 25°C, while the situation was reversed at 15°C. *Caenorhabditis *were not detected in soils, other than under the rotting fruit, or from rotting fruit yet to fall from the trees, but were isolated from molluscs and arthropods associated with the rotting food sources. It remains to be tested whether these are transport hosts for the nematodes. Importantly, it was possible to return to the same site repeatedly and recover animals each time.

## The world of, and in, the worm

So, rather than the monoxenic environment of the agar plate, it is rich habitats like a rotting apple that *C. elegans*' chemosensory system has evolved to process. *C. elegans *lives in a complex ecosystem of bacteria, fungi, slime moulds, hexapod arthropods (adults and larvae), mites, isopods, millipedes, pulmonate molluscs, lumbricid earthworms and other nematode species exploiting this seasonal resource.

*C. elegans *has a fully functioning immune system, with both anti-cellular (bacterial, fungal) and anti-viral arms intact. In the laboratory, in the absence of knowledge of natural pathogens, these systems have been challenged with exotic ones, such as the agents of human disease. *C. elegans *can be killed by many bacterial species, sometimes through direct toxic effect but also via interference with efficient processing of food [8]. In the laboratory one of the hallmarks of pathogenic interaction between a bacterium and *C. elegans *is the proliferation of bacteria within the gut. The species that do this avoid lysis in the nematodes' pharyngeal grinder, and are resistant to digestion. Interestingly, Félix and Duveau [4] found many instances of apparently healthy nematodes with distended intestines full of bacteria and yeasts. Whether this is a nutritive (*C. elegans *is deficient in sterol synthesis and must obtain sterols from food: this may be derived from yeasts) or a morbid interaction remains to be tested. However, fungal pathogens were detected, including some species that produce invasive spores, and others that make nematode-trapping rings and adhesive hyphal traps. As previously described by Félix and colleagues, many nematodes were infected with microsporidia [9], and the first-ever nematode viruses were described from these orchards only last year [10]. Killer bacteria were also isolated, including strains that can digest even the resistant cuticle of the nematodes.

## Field ecology of *Caenorhabditis *species

The findings of Félix and Duveau [4] demonstrate the new kind of science that can now be done with *C. elegans *and *C. briggsae*. Being able to return to a site and reliably resample nematodes makes long-term studies of nematode population genetics possible. The emerging next generation technologies for rapid sequencing of small genomes (the *C. elegans *genome is only 100 Mb) will facilitate complete genetic analysis of individuals and populations [6], permit tracking of polymorphisms through space and time, and help reveal the selective pressures on the whole genome; this will allow measurement of the rates of migration between local populations, permit quantification of the relative rates of outcrossing and selfing, and thus reveal the population genetic structure of *C. elegans *in detail. Similar ecosystems can be surveyed worldwide to identify parallel study sites, and the effects of climate and isolation by distance can be examined. Experimental interventions - for example, introduction of genetically tagged nematodes or manipulation of the ecosystem by selective removal of one class of transport host - are possible in these mesocosm-scale environments. Metagenomic surveys will reveal the interacting populations of microbes cohabiting with the nematodes, and isolate cultures can be tested for positive and negative interactions with nematode genotypes found on the same fruit or isolated elsewhere, revealing genotypic interactions between co-adapted nematode and pathogen genomes. The findings presented by Félix and Duveau may represent the vanguard of a new generation of *C. elegans *researchers: you may find teams of *Caenorhabditis*-ologists paying remarkably close attention to rotting fruit in an orchard near you this summer.

**Figure 1:**
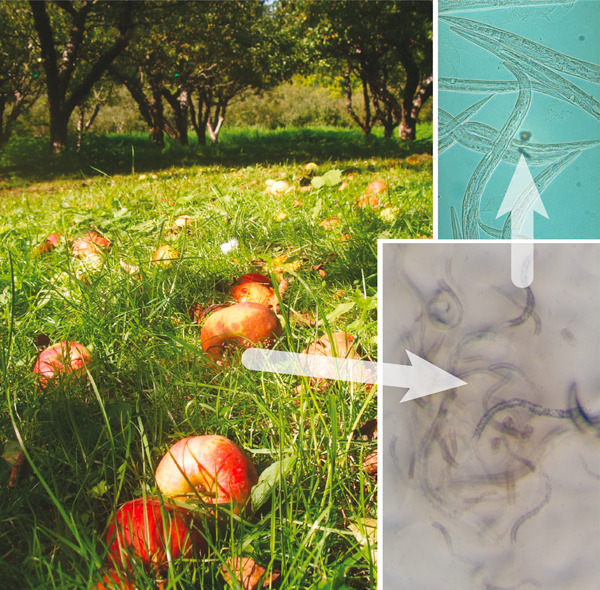
***C. elegans *in the wild**. Félix and Duveau discovered thriving populations of *Caenorhabditis *nematodes (and other organisms) in rotting apples in Orsay orchard, near Paris, France (left image). By isolating these mixed cultures (lower right), and then establishing pure strains of nematode (upper right), they identified both *C. elegans *and *C. briggsae *as common components of the apple rot communities. Images from Marie-Anne Félix (left, lower right) and Mark Blaxter (upper right).
